# Molecular docking analysis of Plasmodium falciparum dihydroorotate dehydrogenase towards the design of effective inhibitors

**DOI:** 10.6026/97320630016672

**Published:** 2020-09-30

**Authors:** Afolabi Owoloye, Ojochenemi A Enejoh, Olusegun M Akanbi, Owolabi M Bankole

**Affiliations:** 1 Parasitology Unit, Department of Animal and Environmental Biology, Adekunle Ajasin University, Akungba-Akoko, Nigeria; 2 Centre for Biocomputing and Drug Development, Adekunle Ajasin University, Akungba-Akoko, Nigeria; 3 Department of Chemistry, Adekunle Ajasin University, Akungba-Akoko, Nigeria

**Keywords:** Plasmodium falciparum, dihydroorotate dehydrogenase, rosmarinic acid, catechin, deoxykaempferol, chloroquine

## Abstract

Malaria remains a global public health burden with significant mortality and morbidity. Despite the several approved drugs available for its management, the parasite has developed resistance to virtually all known antimalarial drugs. The development of a new drug that can combat resistant to Artemisinin based Combination Therapies (ACTs) for malaria is imperative. Plasmodium falciparum dihydroorotate dehydrogenase (PfDHODH), a flavin-dependent mitochondrial enzyme is vital in the parasite's pyrimidine biosynthesis is a well-known drug target. Therefore, it is of interest to document the MOLECULAR DOCKING analysis (using Maestro, Schrodinger) data of DIHYDROOROTATE DEHYDROGENASE PfDHODH from P. falciparum towards the design of effective inhibitors. The molecular docking features of 10 compounds with reference to chloroquine with PfDHODH are documented in this report for further consideration.

## Background

Malaria represents a major peril to world health, infecting between 220 and 300 million people annually, and caused 405 000 mortalities worldwide in the year 2018 [1]. This global disease is caused by parasitic protozoan (Apicomplexan) of the Plasmodium species and is transmitted to humans by the female Anopheles mosquito [2]. Out of the five species of parasite that infect humans, P. falciparum is accountable for the majority of morbidities and mortalities [3] There is no other parasitic infection that has such a wide-ranging influence on human wellbeing. Its persistence has predisposed the evolution of the human genome as underscored by genetic polymorphisms that have ascended by conferring protection against austere malaria [4] Many anti-malarial drugs are in clinical use, nonetheless the development of resistance to both chloroquine and other first-line therapeutics is responsible for the increase in the number of fatalities due to the disease [2,5]. More so, drug resistance has been reported to virtually all known anti-malarial drugs, highlighting the ease by which parasite populations can acclimatize and survive. The resistance of P. falciparum to Artemisinin-based Combination Therapies (ACTs) demonstrated as delayed parasite clearance and linked to Kelch-13-propeller protein polymorphisms has emerged in South East Asia and is hostile to disrupt malaria control efforts [6]. Consequently, there is a pressing need for the development of new antimalarial drugs that can control the infection and can also eliminate the multi-drug resistant P. falciparum. There are some enzymes (proteins) that play a vital role in the survival and proliferation of this parasite; e.g. P. falciparum dihydroorotate dehydrogenase (PfDHODH), P. falciparum hexoses transporter 1 (PfHT1), etc.The flavoenzyme dihydroorotate dehydrogenase (DHODH) [7], is the fourth enzyme in de-novo synthesis of pyrimidine that catalyzes the oxidation of dihydroorotate (DHO) to orotate (ORO). The biosynthesis of de-novo pyrimidine represents a striking and potentially selective target for the development of new drugs against P. falciparum. Unlike human cells, which can both biosynthesis and recover pyrimidine bases, P. falciparum lacks any pathway for the salvage of preformed pyrimidine bases and/or nucleosides and relies entirely on a de novo biosynthesis pathway [7]. Pyrimidines are indispensable metabolites that are precursors for DNA and RNA biosynthesis [8]. Cells obtain pyrimidines either through de novo biosynthesis starting from ammonia (from L-glu), bicarbonate, and L-asp, or by recovering preformed pyrimidine bases (uracil, cytosine, and thymine) or nucleosides (uridine, thymidine, and cytidine). Plasmodium species generally lack pyrimidine salvage enzymes, the de novo pathway serves as the only source of pyrimidines for cell growth. DHODH has been reported as an important target protein identified by high throughput screening of chemical libraries [9]. The inhibition of PfDHODH enzyme would terminate the pyrimidine synthesis pathway. This makes the enzyme a good target for the development of new anti-malarial therapeutics. In this paper, we report the use of high-throughput screening technology of more than 1,000 small drug-like molecules from seven (7) plants to identify several potent and selective inhibitors of the P. falciparum DHODH.

## Methodology

### PfDHODH Structure:

X-ray crystal structure of the P. falciparum dihydroorotate dehydrogenase (PfDHODH) was downloaded from RCSB (Research Collaboratory for Structural Bioinformatics (http://www.rcsb.org) Protein Data Bank (PDB ID: 6GJG) and processed using the Maestro v11.1 interface of Schrodinger (Schrodinger, 2017) following standard procedures where required.

### Ligand Data:

Thousands of phytochemicals from plants of interests (Azadirachta indica, Magniferaindica, Anacardium occidentale, Carica papaya, Oscimum graticimum and Moringa oleifera) were downloaded from the NCBI pubchem databases in 2d (sdf) format to generate a library of compounds for this study. (https://pubchem.ncbi.nlm.nih.gov/). The ligands generated were prepared using the LigPrep interface in the Schrodinger suite (Schrodinger, 2017) with an OPLS3 force field, at pH 7±2 using Epik followed by Lipinski's filter [10].

### Receptor Grid:

A receptor grid was generated in 6GJG using Glide of Maestro v11.1 interface of Schrodinger with default parameters. This estimates the area around the active site in term of co-ordinates x, y and z (6.83, 32.41 and 36.33), respectively.

### Virtual Screening and Molecular Docking:

Molecular docking of ligands with PfDHODH was completed using Schrodinger 11.1 following standard procedures. XP GScore was used for ranking [11]. The ligand interaction interface of Schrodinger 11.1 was used to view the 2D diagram of the ligand binding with the amino acid residues at the active site of the target protein.

### ADME/Tox Analysis:

QikProp module of Maestro 11.1 interface of Schrodinger was used to evaluate the ADMET (Absorption, Distribution, Metabolism, Excretion and Toxicity) properties (SMDDS, 2017) of the lead compounds. Various physio-chemical descriptors were calculated to further account for the potential of the lead molecule to act as efficient drug candidate.

### Validation of Molecular Docking Result:

The bioactivities of the target protein from the database was 584 IC_50_, the conical smiles of IC_50_ were downloaded. The conical smiles file was open with 'Number' (Macbook pro 2016) to view the properties of the file followed by cleaning of the data. The file was saved in comma separated value (.csv) format. The csv file was converted in to 2d (sdf) format using DataWarrior v.5.0 (2019). The converted 2D (sdf) file was opened using Schrodinger 11.1 (2017-1), the file was prepared using ligprep (pH: 7±2, forcefield: OPLS3). Ligand docking interface of Schrodinger 11.1 (2017-1) was used to dock the prepared ligands using glide of target protein receptor with extra precision (XP) algorithm. A plot of the docking score of randomly selected 101 compounds was plotted against their respective pIC_50_ value (PCHEMBL VALUE). Spearman correlation coefficient (R) of the graph was calculated.

## Results and Discussion:

Malaria parasite has evolved drug resistance against virtually all known anti-malarial drugs. The efficacy of anti-malarial drugs is waning due to the ability of Plasmodium species to develop drug resistance. Mechanisms of resistance of P. falciparum against various antimalarial drugs is analyzed using genetic, molecular and biochemical approaches which have shown that mutations of the P. falciparum multidrug resistant protein 1 (Pfmdr1) and P. falciparum chloroquine resistance (Pfcrt) gene. The latter has led to the impairment of chloroquine uptake by the parasite vacuole. [12]. The protein selected for this in-silico study was obtained from literature, it is present in the vital metabolic pathways of P. falciparum. The protein (enzyme) is essential for survival of the parasite. The library of compounds was screened against the protein using Ligand Docking Tool on Schrodinger 11.1. Ligands with the best hit and docking score with this protein were selected. Chloroquine docking score was set as standard score, all the ligands with docking score below (in the negative) the standard were screened out. For PfDHODH, more than fifty compounds were found to have high binding scores than the co-crytallized ligand in the active site, the best ten (10) compounds were selected using ADMETox. The reference compound, chloroquine had total interaction energy at -5.03 kcal/mol, which was lesser than the total interaction energy of the lead compounds. This could have been as a result of the greater and better interaction of the prime compounds with the target protein. This comparison shows that the prime compounds identified against malaria had better inhibition than already known inhibitor present in the crystal structure of PfDHODH.

## Interaction Profile:

A significant characteristic of P. falciparum is its ability to undergo vast re-organization of genetic make-up during the course of its life cycle in several host environments. Inhibition of essential metabolic enzymes can be disadvantageous for the parasite's survival, one of which is the PfDHODH. This enzyme belongs to the β/α-barrel structural fold class and binds in a site between the two N-terminal α-helices (starting at amino acid Gly181) and the body of the barrel domain. The binding site of the protein (PfDHODH) is adjacent to the flavin mononucleotide (FMN) cofactor and is largely hydrophobic in nature. However, no structural data are available for the ligands with the highest docking score bound to DHODH from any species. In this study, the flavonoid group of the inhibitor is bound in an entirely hydrophobic pocket where it is in H-bond contact with Gly181, Hie185, Phe188, Arg265, Tyr528, Leu531 and Val532, and where it forms edge-to-face stacking interactions. The PfDHODH active site has two components, vis-a-vis the hydrogen-bond site between His185 and Arg256 (and nearest to FMN) and the adjacent hydrophobic pocket that is lined with amino acid residues in part played by helices 1 (amino acids 162-176) and 2 (amino acids 181-194) [13,14]. In this study, rosmarinic acid had the highest docking score -11.545 kcal/mol. Rosmarinic acid is an ester of caffeic acid and 3, 4-dihydroxyphenyllactic acid. It is commonly found in species of the Boraginaceae and the subfamily Nepetoideae of the Lamiaceae.

**Figure 1:**
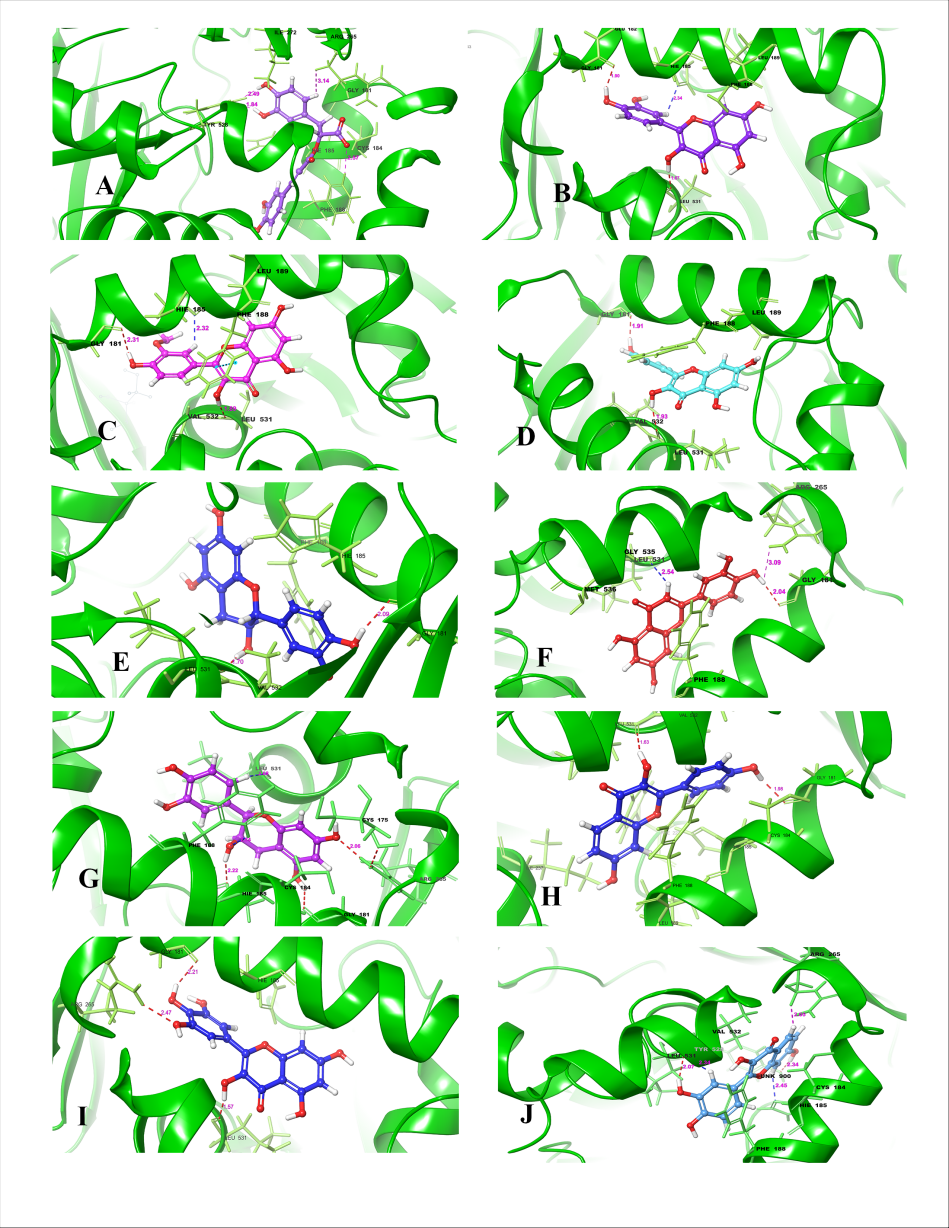
3D structure of interaction of PfDHODH with (A) Rosmarinic acid, (B) Quercetin, (C) Isorhamnetin, (D) Kaempferol, (E) Catechin, (F) Luteolin, (G) Epicathecin, (H) Deoxykaempferol, (I) Myricetin and (J) Fesitin

Rosmarinic acid (Figure 1A) forms H-bond with four (4) amino residue of PfDHODH (Arg265:1.73 Å, Phe188:2.57 Å, Tyr528: 1.84 Å and 2.48 Å). Arg256 donates a proton via C11, the measured distance of all the H-bonds were <2.50A, this show a greater affinity. One of the atoms within a short distance of the backbone carbonyl of Arg265 (distance of 1.73 Å) is the optimal angle (<90°) to the target protein axis to maximize a favourable binding interface of the 'H-bond'. Auffinger [15] reported similar optimal angle that favours interaction with the delocalized electrons of the Gly535-Met536 amide. Delocalized electron of Arg265-Cys175 was also observed, bridging N atom forms a good hydrogen bond (2.6 Å) to Cys175 (Figure 1A). The diphenol of the second benzene ring is within hydrogen-bonding distance of any amino acid in the protein and its closest contact is with the backbone amide of Tyr528 (distance of 1.84 and 2.48 Å).

Catechin is the (+)-enantiomer of catechin and a polyphenolic antioxidant plant metabolite. It is a flavocoxid, consisting of plant derived flavonoids which have anti-inflammatory activity and are used to treat chronic osteoarthritis [16]. The binding mode of catechin (Figure 1E) was compared with that of rosmarinic acid, as well as either other compounds that inhibit PfDHODH with similar potency in this study (Table 1 - see PDF). All inhibitors for which structural data are available occupy the hydrogen-bond site, making hydrogen-bond contacts with Gly181 (2.09 Å), leu531 (1.70 Å) and Val532 (1.7 Å). The docking score of (+)-catechin was -11.525 kcal/mol. Thus, the cofactor binding site in the crystal structure of PfDHODH i.e., the F1T binding pocket, along with adjacent solvent exposed cavities, were used as receptor grids to dock using Glide [11].

All the ten compounds make close hydrogen-bond contacts with the amino residue(s) of PfDHODH (Figs 1A-1J), although the position of binding amino residue is rotated in each structure to allow optimal geometry for the individual interactions. However, unlike deoxykaempferol, quercetin (Figure 1B) and isorhamnetin (Figure 1C) do not form a hydrogen-bond interaction with Arg265 because the amino acid residue is rotated away from the ligand in order to accommodate other H-bond. Nevertheless, it is possible that an electrostatic interaction between Arg265 and the -OH group of the ligands contribute to the binding energy, and thus interactions with Arg265 is likely to be important to high-affinity binding of the ten inhibitors. The importance of the binding interactions with both His185 and Arg265 is supported by site-directed mutagenesis studies, where we found that mutation of either residue to Ala reduced the binding affinity of PfDHODH for several characterized triazolopyrimidines [13,17].

**Figure 2:**
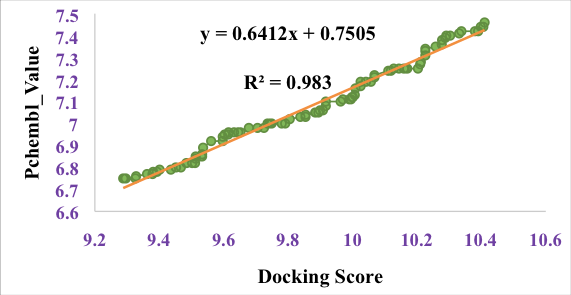
Correlation graph between the PfDHODH pIC50 and docked scores. R2: correlation coefficient 0.983

**Figure 3:**
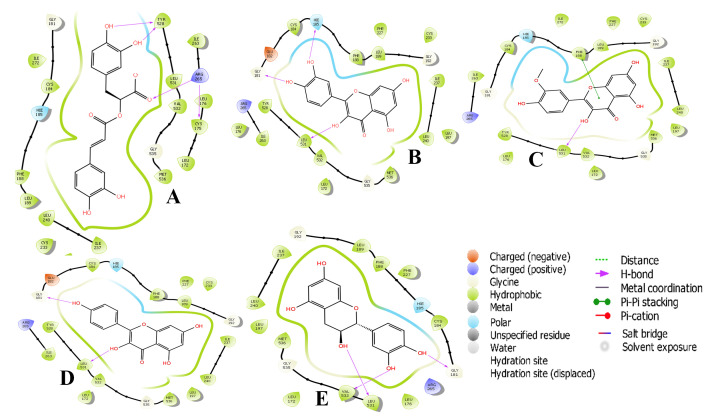
2D structural representation of PfDHODH interaction with (A) Rosmarinic acid, (B) Quercetin, (C) Isorhamnetin, (D) Kaempferol, (E) Catechin

**Figure 4:**
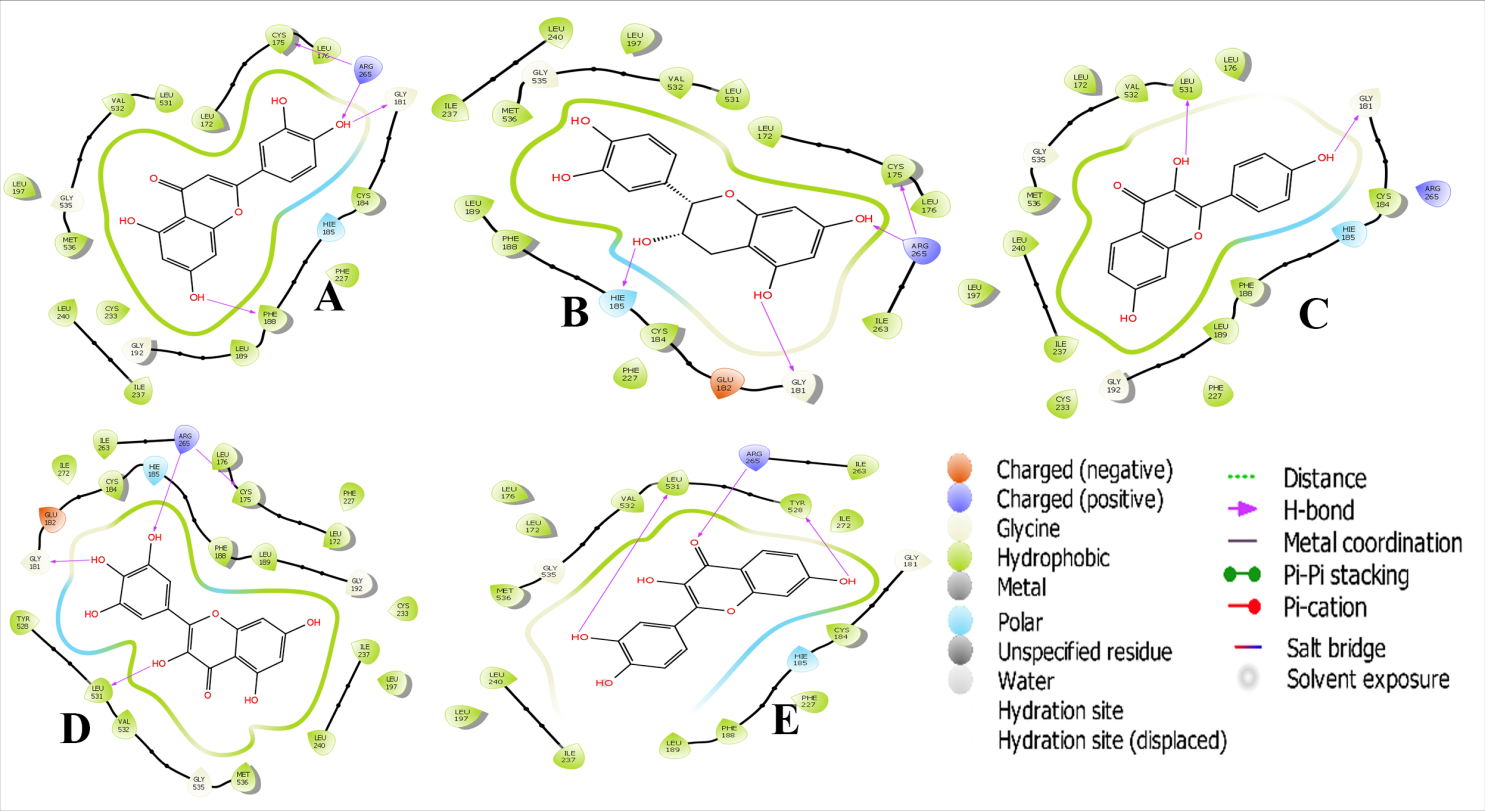
2D structural representation of PfDHODH interaction (A) Luteolin, (B) Epicathecin, (C) Deoxykaempferol, (D) Myricetin (E) Fesitin.

Fisetin is a 7-hydroxyflavonol with additional hydroxy groups at positions 3, 3' and 4'. It has a role as an antioxidant, an inhibitor, an anti-inflammatory agent, a metabolite and a plant metabolite [18]. Fisetin, in addition to situating itself in a deep and proximate orientation within PfDHODH binding pocket, a highly stable network of hydrogen bonds within <2.5 Å bond distance was also observed (Figure 3). The compound formed strong H-bond with residues His185 (2.45 Å), Arg265 (2.05 Å), Leu531 (2.07 and 2.34 Å) and Tyr528 (2.34 Å). These residues have been reported to be essential in inhibition of PfDHODH [19]. The third benzene ring (R3) in fisetin, which harbours two OH- groups efficiently, twists optimally to engage in a hydrogen trade-off with catalytic important Leu531, thus disrupting the pocket's alignment. Our knowledge about the network of hydrogen outside the hydrophobic shell is in coherence with a recent report [20], where little evidence was provided about the lower hydrophobic shell. In exploring this, we discovered that rings 1 and 2 are buried deeply within the hydrophobic pocket binding with Arg265 (2.05 Å) and Tyr528 (2.34 Å) (Figure 4). Epicatechin is a flavonoid, occurring especially in woody plants, it is a catechin with (2R,3R)-configuration. It has a role as an antioxidant [21]. It binds easily to bacterial proteins, blocking bacteria from adhering to cell walls and disrupting their ability to destroy them [21]. In this study epicatechin was observed to be buried deep within the hydrophobic pocket of the target forming H-bond and a water bridge between Arg265 and Cys175. The H-bond distance observed in the first benzene ring (R1) with two OH- was less than 2.5 Å. This study revealed key amino acid residues necessary for ligand binding. Residues of PfDHODH, particularly, Hie185, Arg265 and leu531, have been reported to be crucial for ligand binding [22]. Interestingly, the ligands with high docking scores in this study were found interacting with these key residues at the target's active site. Docking the protein with known co-crystalized inhibitor using Ligand Docking Tool on Schrodinger 11.1 validated the crystal structure of PfDHODH and the RMSD (Root Mean Square Deviation) value was confirmed as <2, thus validating these computational tools. Furthermore, the graph of experimentally determined pIC_50_ (pChembl-value) of PfDHODH against the docking score (Figure 2) showed a good correlation (R2=0.983). This proved that the in-silico experiment can be reproduced either by in-vitro or in-vivo experiment.

## ADME/Tox Properties:

The ten selected compounds were found to follow Lipinski's rule of five: ADMET properties and bioactivity scores of compounds are showed in Table 1 (see PDF). The hit compounds were shown to follow Lipinski's rule of 5, which underscores the compounds as potent drug candidates. The screening of ligands (compounds) using Absorption, Distribution, Metabolism and Elimination (ADME) describes the efficiency, efficacy, and ability of the ligands to reach its site of action and to be easily excreted (eliminated) from the body. The Lipinski rule of five recapitulates the molecular properties of compound to be orally active and druggable. The rule permits hydrogen bond donor's ≤5, hydrogen bond acceptors ≤10, molecular weight <500Da, and octanol-water partition coefficient (logP) <5. In this study, the hit compounds violated none of the lipinski's of five, this makes the hit compounds potential antimalarial drug candidates.

## Conclusion

The molecular docking features of 10 compounds with reference to chloroquine with PfDHODH are documented in this report for further consideration.
